# Questionnaire survey investigation of the present status of dietetic consultation at community pharmacies from the perspectives of registered dietitians and pharmacists

**DOI:** 10.1186/s12913-021-06959-3

**Published:** 2021-09-08

**Authors:** Hayato Kizaki, Tomu Ota, Saki Mashima, Yoshimi Nakamura, Shoko Kiyokawa, Hidenori Kominato, Hiroki Satoh, Yasufumi Sawada, Satoko Hori

**Affiliations:** 1 Division of Drug Informatics, Keio University Faculty of Pharmacy, 1-5-30 Shibakouen, Minato-ku, Tokyo, 105-8512 Japan; 2 I&H Co., Ltd., 18 Omasucho, Ashiya, Hyogo 659-0066 Japan; 3 Graduate School of Pharmaceutical Sciences, The University of Tokyo, 7-3-1 Hongo, Bunkyo-ku, Tokyo, 113-0033 Japan

**Keywords:** Community pharmacy, Registered dietitian, Pharmacist, Dietetic consultation, Cooperation

## Abstract

**Background:**

Registered dietitians are rarely employed at community pharmacies in Japan, even though dietetic advice might benefit some patients.

**Objective:**

To clarify the present status of dietetic consultation provided by registered dietitians and their collaboration with pharmacists in community pharmacies.

**Methods:**

We conducted a cross-sectional questionnaire-based survey of pharmacists and registered dietitians who work in community pharmacies. The surveyed items were: frequency of dietetic consultation, awareness of one’s knowledge and ability to conduct dietetic consultation, concerns, pharmacists’ recognition of the need for nutritional support at community pharmacies, and cooperation between registered dietitians and pharmacists.

**Results:**

Sixty-six registered dietitians, 53 pharmacists in pharmacies with registered dietitians/dietitians, and 110 pharmacists in pharmacies without registered dietitians/dietitians responded. The frequency of dietetic consultation regarding obesity and hypertension was significantly higher for registered dietitians than for pharmacists. The ability to conduct dietetic consultation regarding diseases/conditions such as kidney disease not requiring dialysis, hyperuricemia, gout, obesity and hypertension was also significantly higher for dietitians than pharmacists. More than 70% of pharmacists recognized the importance of nutritional support at community pharmacies, while 56.1% of registered dietitians noted that they were not able to fully utilize their occupational abilities. Registered dietitians were divided into two groups: registered dietitians who answered that they were able to utilize their occupational abilities and those that answered they were not. The former group was more likely to ask pharmacists about patients’ medication for dietetic consultation and to be asked to provide dietetic consultation to patients. The latter group was more likely to find difficulty in scheduling dietetic consultation.

**Conclusion:**

Our results suggest that registered dietitians in community pharmacies have a greater explanatory ability than pharmacists concerning nutritional and dietary management for patients. It may be important for pharmacists to improve cooperation with registered dietitians by providing more opportunities for dietetic consultation.

**Supplementary Information:**

The online version contains supplementary material available at 10.1186/s12913-021-06959-3.

## Background

The Ministry of Health, Labor and Welfare in Japan launched the “Pharmacy Vision for Patients” in 2015 [1]. In the statement, community pharmacies are required not only to manage medication information in an integrated and continuous manner, but also to provide health support to residents. The functions of pharmacies in supporting the health of residents include counseling on medications, recommendations to see a doctor, and nutritional management. These functions of pharmacies can contribute to the management and promotion of health from the perspective of both treatment and prevention. However, pharmacist training does not usually include specialized education on nutrition, and pharmacists may find it difficult to provide appropriate dietetic consultations for patients with various diseases and health conditions.

The profession of registered dietitian is a national qualification in Japan, and registered dieticians have the potential to contribute to health support in community pharmacies by providing highly specialized nutrition care for patients. A registered dietitian is defined as “a person who is licensed by the Minister of Health, Labor and Welfare and who […] is engaged in providing nutritional guidance necessary for the medical treatment of the sick and injured, etc.” (Dietitians Act, Article 1). The title of “dietitian” can be obtained by graduating from a dietitian training facility, but to receive the title of “registered dietitian”, it is required to pass a national exam after a certain period of work experience and/or practical training. There are approximately 250,000 licensed registered dietitians [2], who work in various fields where nutrition management is necessary, such as medical care, welfare, sports, and local government. In the medical field, registered dietitians mainly work in hospitals and other medical facilities, but their role in community pharmacies is currently very limited. It has been reported that continuous nutritional intervention after discharge improves the nutritional state of patients with pneumonia and reduces the rate of rehospitalization [3]. A study in Japan found that intervention by dietitians to limit salt intake led to a reduction of both salt intake and blood pressure [4]. It has also been found that home nutritional guidance by a multidisciplinary team including registered dietitians contributed to maintaining the enjoyment of eating for diabetic nephropathy patients [5]. Thus, nutritional guidance by registered dietitians is considered to be important in terms of improving patients’ condition and preventing deterioration. Indeed, as community pharmacies are accessible to all, utilizing the capabilities of registered dietitians in community pharmacies should contribute to the management and promotion of the health of local residents from the perspective of both treatment and prevention.

Among the dietetic consultations provided by registered dietitians, those conducted in hospitals mainly involve guidance such as setting up specific menus based on the physician’s orders through dietary prescriptions. The charges for these consultations are included in the medical fees. The targeted persons for dietetic consultation, for whom such charges can be reimbursed, are limited to “patients requiring special diets specified by the Minister of Health, Labor and Welfare, cancer patients, patients with impaired eating or swallowing functions, or patients with malnutrition”. Meta-analyses indicate that dietetic consultations in hospitals provided by registered dietitians are effective in the treatment and prevention of diseases [6, 7].

On the other hand, dietetic consultations provided by registered dietitians in community pharmacies do not need to be requested by physicians, and charges would not be reimbursed. These consultations would be considered as a part of the pharmacy’s service. Dietetic consultations are often provided free of charge as a part of health support provided by pharmacies. These activities are considered to have beneficial effects such as promoting sales of health foods and other health support products and increasing the number of customers at the pharmacies. There are no rules regarding the targeted persons for such dietetic consultations, and registered dietitians are able to provide nutritional care for all, including healthy persons. Thus, dietetic consultations provided by registered dietitians in community pharmacies can play an important role in heath support to residents. However, there is little evidence regarding the effectiveness of dietetic consultations by registered dietitians in community pharmacies. Furthermore, an investigation by the Ministry of Health, Labor and Welfare in 2018 revealed that the number of pharmacies in which registered dietitians work was less than 10% of the total [8].

Therefore, it is important to clarify the implementation status of dietetic consultations provided by registered dietitians in community pharmacies, as well as the problems registered dietitians face and how they work in cooperation with pharmacists, in order to obtain suggestions for promoting and expanding the role of registered dietitians in community pharmacies in Japan. The purpose of this study was to clarify the current status of dietetic consultation provided by registered dietitians and their collaboration with pharmacists in community pharmacies.

## Materials and method

### Design and participants

In this study, self-administered questionnaire survey was conducted anonymously. The questionnaires were sent to the 74 registered dietitians and 195 supervising pharmacists working at 195 community pharmacies operated by I&H Co., Ltd. The included community pharmacies, widely distributed throughout Japan, mainly in the *Kanto* and *Kansai* areas. A questionnaire and a document explaining the study were sent to each pharmacy from the Faculty of Pharmacy, Keio University. The document stated that the survey was conducted confidentially and anonymously. Subjects who read the document and agreed to cooperate in this study were asked to complete the questionnaire survey on paper. The completed questionnaires were returned directly from the individuals to the Faculty of Pharmacy, Keio University. It was clearly stated in the document that no one from I&H Co., Ltd. would have access to the contents of individual responses. Questionnaires returned between July 8, 2019 and July 31, 2019 were used in the analysis.

### Questionnaire survey

The questionnaire used multiple-choice and descriptive questions in Japanese. The questions and options in the questionnaire were developed based on observation of registered dietitians’ work in community pharmacies and advice from registered dietitians working in pharmacies. In the present survey, we ensured the relevance to practice of the questionnaires by reflecting opinions of several registered dietitians working at community pharmacies in the questionnaire before the questionnaire survey. Three types of questionnaires were prepared: one for registered dietitians, one for pharmacists in community pharmacies where registered dietitians or dietitians work, and one for pharmacists in community pharmacies where neither registered dietitians nor dietitians work (translations of the questionnaires are given as an Additional file 1).

Registered dietitians and pharmacists were asked in common about the frequency of dietetic consultation with patients with various diseases or symptoms using a 5-point scale, with “1; Never”, “2; Rarely”, “3; Neither”, “4; Sometimes”, and “5; Often”, and also on how much they could explain about nutrition and diet that needed attention using a 5-point scale, with “1; Never”, “2; Not very well”, “3; Neither”, “4; Somewhat”, and “5; Very well” for each disease.

Only pharmacists were asked about their perceptions of nutritional management in community pharmacies using a 5-point scale, with “1; Strongly disagree”, “2; Disagree a little”, “3; Neither”, “4; Agree a little”, and “5; Strongly agree”. Pharmacists working in community pharmacies with registered dietitians/dietitians were asked in descriptive questions if they were satisfied with the presence of registered dietitians/dietitians in their pharmacies and what challenges pharmacies face to fully utilize the profession of registered dietitians/dietitians.

Only registered dietitians were asked in multiple-choice questions about the timing and/or situation that led to dietetic consultation, and whether they were able to utilize their occupational abilities. They were also asked about difficulties or challenges of dietetic consultation using a 5-point scale, with “1; Strongly disagree”, “2; Disagree a little”, “3; Neither”, “4; Agree a little”, and “5; Strongly agree”, and about the frequency of cooperation between registered dietitians and pharmacists using a 5-point scale, with “1; Never”, “2; Rarely”, “3; Neither”, “4; Sometimes”, and “5; Often”. In addition, they were asked about challenges and solutions regarding dietetic consultation in community pharmacies in descriptive questions.

### Statistical analysis

Registered dietitians were classified into two groups according to the extent to which they were able to utilize their occupational abilities as registered dietitians: the “Not able to fully utilize occupational abilities” group and the “Able to utilize occupational abilities” group. Those who answered “never” or “not very well” were classified as the former, and those who answered “somewhat” or “very well” were classified as the latter, in the question asking whether they were able to utilize their abilities as registered dietitians. For these two groups, “difficulties or challenges in dietetic consultation” and “the frequency of cooperation between registered dietitians and pharmacists” were cross-tabulated. The χ-square test was used to evaluate the difference between the two groups.

For questions common to registered dietitians and pharmacists, the responses (five options) were scored, and the scores were compared among three groups: registered dietitians (D), pharmacists working in a community pharmacy with a registered dietitian/dietitian (P with D), and pharmacists working in a community pharmacy without registered dietitian/dietitian (P without D). The Kruskal-Wallis test was performed at the 5% level of significance, and where differences were found, the Dunn-Bonferroni method was used to compare those groups. The statistical analysis software IBM®SPSS®Statistics version 27 and Microsoft Office Excel were used for these analyses.

Moreover, the contents of the free text responses were subjected to quantitative text analysis using the free software KH Coder [9]. For each question, words with high frequencies of occurrence were extracted as characteristic words. A co-occurrence network was also created to show the strength of association between featured words.

## Results

### Background Information of respondents

The respondents were 66 registered dietitians (response rate; 89.2%), 53 pharmacists in community pharmacies where registered dietitians or dietitians work (response rate; 79.1%), and 110 pharmacists in community pharmacies where neither registered dietitians nor dietitians work (response rate; 85.9%). The background information of the respondents is summarized in Table 1. Most of the registered dietitians were in their 20’s or 30’s, and all were females. The most common age range of pharmacists with or without registered dietitian/dietitian was the 30’s, and about 60% of them were female. Most of the registered dietitians had less than 1 year of experience, while most of the pharmacists had more than 3 years of experience.

**Table 1:** Characteristics of respondents

Characteristics	Registered dietitian *n* = 66	Pharmacist with registered dietitian/dietitian *n* = 53	Pharmacist without registered dietitian/dietitian *n* = 110
Age			
20’s	60	16	13
30’s	6	24	52
40’s	0	9	27
50’s	0	3	13
60 years or more	0	1	5
Gender			
Male	0	22	49
Female	66	31	61
Years of experience			
Less than 1 year	32	0	0
1–3 years	18	0	1
3–5 years	7	11	13
5–10 years	7	23	30
10 years or more	0	19	66
No answer	2	0	0

### Implementation of dietetic consultation by registered dietitians

Dietetic consultation in this study was defined as providing any guidance based on nutrition science to improve nutritional condition. There were two types of dietetic consultation: “ordinary dietetic consultation” and “dietetic consultation sessions”. Dietetic consultation sessions were held on days when registered dietitians were available to provide dietetic consultation on a full-time basis, and users of the pharmacy could make an appointment in advance to consult with a registered dietitian individually about their dietary and nutritional concerns. All dietetic consultations that did not meet the definition of “dietetic consultations sessions” were defined as “ordinary dietetic consultations”.

The most common reasons that led to ordinary dietetic consultation were when patient consulted while waiting for medication to be prepared or when the pharmacist recommended the patient consult a registered dietitian (Table 2). Only about half of the registered dietitians provided dietetic consultation by approaching the patients themselves.

**Table 2:** Timing and/or situation that triggered dietetic consultation

Item	Number (%)
A patient consulted at the time of presenting prescription	11 (25.6)
A patient consulted while waiting for medication	33 (76.7)
A patient consulted at the time of payment	27 (62.8)
A visitor who did not have a prescription consulted	15 (34.9)
Registered dietitian approached the patient	22 (51.2)
Pharmacist recommended the patient to consult registered dietitian	33 (76.7)
Others	3 (7.0)

Numbers in parentheses indicate the percentage out of the 43 respondents who conducted dietetic consultation in daily work

A quantitative text analysis of registered dietitians’ responses to issues related to ordinary dietetic consultation at community pharmacies showed that words such as “time,” “busy,” and “office work” were among the top responses. The actual responses to the questionnaire included, “I can not find time to do it while doing administrative work,” and “I have to prioritize my office work when I am busy, so I can not provide enough consultation”.

For the following questions, “dietetic consultation sessions” and “ordinary dietetic consultation” were treated as “dietetic consultation” without distinguishing between them.

### Frequency of dietetic consultation for each disease in each profession, and ability to explain about nutrition and dietary intake for each disease

The frequency of dietetic consultation with patients with each disease or symptom and how much could be explained about nutritional and dietary factors that need attention were compared among three groups (D, P with D, P without D). Regarding the difference of frequency between diseases, registered dietitians provided dieteteic consultation significantly more often than pharmacists for patients with obesity and hypertension (Table 3). Registered dietitians also provided dietetic consultation significantly more often than P without D for diabetic patients. Regarding how much could be explained about nutritional or dietary factors that need attention, registered dietitians had a significantly higher ability than pharmacists for patients with kidney disease not requiring dialysis, hyperuricemia, gout, obesity, and hypertension (Table 4).

**Table 3:** Frequency of dietetic consultation for patients with various diseases

Disease	Group	Never (%)	Rarely (%)	Neither (%)	Sometimes (%)	Often (%)
Kidney disease not requiring dialysis	D	31.8	12.1	21.2	18.2	13.6
	P with D	18.9	28.3	22.6	28.3	0.0
	P without D	22.7	32.7	15.5	23.6	2.7
Kidney disease in need of dialysis	D	45.5	16.7	22.7	9.1	3.0
	P with D	34.0	32.1	20.8	11.3	0.0
	P without D	45.5	29.1	8.2	10.9	2.7
Liver disease	D	40.9	18.2	22.7	10.6	3.0
	P with D	18.9	47.2	22.6	9.4	0.0
	P without D	26.4	38.2	20.0	10.9	0.9
Diabetes*	D ^*b*^	12.1	3.0	12.1	18.2	48.5
	P with D	1.9	3.8	7.5	64.2	20.8
	P without D ^*b*^	1.8	13.6	16.4	49.1	18.2
Peptic ulcer	D	34.8	19.7	24.2	12.1	4.5
	P with D	24.5	37.7	20.8	13.2	1.9
	P without D	30.0	38.2	17.3	12.7	0.0
Anemia	D	25.8	10.6	24.2	28.8	6.1
	P with D	9.4	32.1	20.8	30.2	3.8
	P without D	15.5	28.2	30.0	22.7	2.7
Dyslipidemia*	D	18.2	1.5	13.6	25.8	37.9
	P with D	3.8	5.7	13.2	52.8	22.6
	P without D	7.3	10.9	18.2	50.0	10.9
Hyperuricemia, gout	D	24.2	6.1	16.7	39.4	9.1
	P with D	3.8	24.5	28.3	34.0	7.5
	P without D	13.6	20.0	22.7	35.5	6.4
Heart disease	D	42.4	21.2	19.7	10.6	3.0
	P with D	22.6	49.1	20.8	5.7	0.0
	P without D	34.5	37.3	20.9	5.5	0.0
Obesity*	D ^*a, b*^	18.2	1.5	18.2	27.3	31.8
	P with D ^*a*^	15.1	24.5	18.9	34.0	5.7
	P without D ^*b*^	23.6	26.4	20.9	21.8	6.4
Hypertension*	D ^*a, b*^	16.7	0.0	10.6	18.2	50.0
	P with D ^*a*^	3.8	13.2	22.6	49.1	9.4
	P without D ^*b*^	6.4	19.1	18.2	48.2	7.3
Constipation	D	21.2	16.7	24.2	16.7	18.2
	P with D	5.7	26.4	24.5	37.7	3.8
	P without D	11.8	25.5	20.9	35.5	4.5
Diarrhea	D	31.8	36.4	19.7	4.5	4.5
	P with D	22.6	28.3	28.3	18.9	0.0
	P without D	27.3	37.3	18.2	13.6	1.8
Cancer	D	53.0	13.6	21.2	9.1	0.0
	P with D	39.6	32.1	20.8	5.7	0.0
	P without D	52.7	25.5	12.7	6.4	0.9

D: Registered dietitian

P with D: Pharmacist who works at a pharmacy with a registered dietitian/dietitian

P without D Pharmacist who works at a pharmacy without a registered dietitian/dietitian

* *p* < 0.05 (Kruskal-Wallis test); ^*a,b*^ *p* < 0.05 (Dunn-Bonferroni)

**Table 4:** Ability to explain about nutrition and dietary intake for each disease

Disease	Group	Never (%)	Not very well (%)	Neither (%)	Somewhat (%)	Very well (%)
Kidney disease not requiring dialysis*	D ^*a, b*^	1.5	19.7	24.2	45.5	7.6
	P with D ^*a*^	3.8	45.3	28.3	24.5	0.0
	P without D ^*b*^	6.4	30.0	30.0	33.6	0.9
Kidney disease in no need of dialysis	D	1.5	30.3	37.9	27.3	1.5
	P with D	7.5	50.9	22.6	20.8	0.0
	P without D	15.5	31.8	25.5	27.3	0.9
Liver disease	D	4.5	36.4	34.8	21.2	1.5
	P with D	9.4	47.2	26.4	18.9	0.0
	P without D	11.8	30.0	36.4	20.0	0.9
Diabetes	D	0.0	3.0	10.6	56.1	28.8
	P with D	0.0	1.9	17.0	71.7	11.3
	P without D	0.0	4.5	15.5	62.7	17.3
Peptic ulcer	D	3.0	21.2	39.4	27.3	6.1
	P with D	5.7	30.2	30.2	34.0	1.9
	P without D	6.4	30.0	35.5	24.5	2.7
Anemia	D	0.0	4.5	22.7	59.1	12.1
	P with D	0.0	13.2	20.8	62.3	5.7
	P without D	0.0	16.4	22.7	53.6	8.2
Dyslipidemia	D	0.0	3.0	24.2	47.0	24.2
	P with D	0.0	3.8	28.3	66.0	3.8
	P without D	0.9	8.2	25.5	55.5	10.9
Hyperuricemia, gout*	D ^*a, b*^	0.0	4.5	15.2	59.1	19.7
	P with D ^*a*^	0.0	7.5	28.3	66.0	0.0
	P without D ^*b*^	0.9	11.8	22.7	54.5	10.0
Heart disease	D	12.1	40.9	28.8	15.2	1.5
	P with D	13.2	43.4	35.8	9.4	0.0
	P without D	12.7	34.5	34.5	17.3	1.8
Obesity*	D ^*a, b*^	0.0	6.1	19.7	48.5	24.2
	P with D ^*a*^	3.8	11.3	28.3	49.1	9.4
	P without D ^*b*^	3.6	10.9	30.0	47.3	9.1
Hypertension*	D ^*a, b*^	0.0	1.5	12.1	54.5	30.3
	P with D ^*a*^	1.9	5.7	20.8	67.9	5.7
	P without D ^*b*^	0.0	9.1	17.3	62.7	11.8
Constipation	D	1.5	9.1	24.2	45.5	18.2
	P with D	1.9	11.3	22.6	62.3	3.8
	P without D	0.9	10.0	30.0	50.0	10.0
Diarrhea	D	1.5	21.2	36.4	30.3	9.1
	P with D	1.9	26.4	32.1	39.6	1.9
	P without D	3.6	22.7	34.5	34.5	4.5
Cancer	D	33.3	45.5	15.2	4.5	0.0
	P with D	35.8	26.4	32.1	5.7	1.9
	P without D	32.7	30.0	24.5	11.8	1.8

D: Registered dietitian

P with D: Pharmacist who works at a pharmacy with a registered dietitian/dietitian

P without D: Pharmacist who works at a pharmacy without a registered dietitian/dietitian

* *p* < 0.05 (Kruskal-Wallis test); ^*a,b*^ *p* < 0.05 (Dunn-Bonferroni)

### Pharmacists’ perceptions on nutritional Management in Pharmacies

Regarding pharmacists’ perceptions of nutritional management in community pharmacies, the percentage of subjects who answered “Agree a little”, or “Strongly agree” was calculated for each scene (Table 5). In all categories, more than 70% of the respondents recognized the importance of nutritional management in community pharmacies. In particular, all pharmacists in community pharmacies with registered dietitians/dietitians recognized the need for nutritional and dietary management for patients in the pharmacy.

**Table 5:** Pharmacists’ recognition of nutritional support at pharmacy

Item	P with D (*n* = 53)	P without D (*n* = 110)	*P* value
Nutritional and dietary management at pharmacy is necessary.	100.0%	86.4%	0.005
Nutritional and dietary management at pharmacy improves patients’ QOL.	90.6%	85.5%	0.362
Nutritional and dietary management at pharmacy improves the patient’s laboratory test data such as blood pressure or blood glucose.	88.7%	86.4%	0.679
Nutritional and dietary management at pharmacy lead to reducing the number of medications patients use.	75.5%	68.2%	0.339
The number of pharmacies with registered dietitians will increase in the future.	84.9%	80.0%	0.449

P with D: Pharmacist who works at a pharmacy with a registered dietitian/dietitian

P without D: Pharmacist who works at a pharmacy without a registered dietitian/dietitian

In the free descriptive answers of pharmacists about the merit of having a registered dietitian/dietitian in the pharmacy, the word “guidance” appeared frequently, and “guidance” co-occurred with the words “registered dietitian” or “concrete”. The actual responses to the questionnaire included, “The registered dietitian is able to provide accurate guidance”, or “registered dietitians are able to provide more specific and detailed explanations than pharmacists”. In addition, many pharmacist respondents answered, “pharmacists can provide general dietary guidance but cannot suggest specific recipes, while registered dietitians provide advice that is easy to incorporate into daily life”. The challenges for registered dietitians to be active in community pharmacies included reviewing their work load to enable them to provide dietetic consultation while patients are waiting, and low awareness of the availability of dietetic consultation in the pharmacy.

### Frequency of collaboration with pharmacists and the difficulties and challenges faced by registered dietitians, based on their ability to utilize occupational abilities

Registered dietitians were asked whether they were able to utilize their occupational abilities using a 5-point scale. 56.1% of the respondents answered “not at all” or “not very much”. None of the registered dietitians responded that they utilized their occupational abilities very well. Twelve registered dietitians were classified into the “Able to utilize occupational abilities” group and 37 registered dietitians into the “Not able to fully utilize occupational abilities” group. The frequency of cooperation with pharmacists and the degree of difficulty and anxiety faced by registered dietitians were compared between the two groups.

In the “Able to utilize occupational abilities” group, the number of respondents who answered “I ask the pharmacist about the patient’s medication for dietetic consultation” or “A pharmacist suggests/requests a dietetic consultation by a registered dietitian for patients” was significantly higher than in the “Not able to fully utilize occupational abilities” group (Table 6). In the “Not able to fully utilize occupational abilities” group, the number of respondents who felt that “It is difficult to find opportunity for dietetic consultation” was significantly higher than the other group (Table 7).

**Table 6:** Frequency of interactions between registered dietitians and pharmacists based on the state of utilization of occupational abilities

Item	Not able to fully utilize occupational abilities	Able to utilize occupational abilities	*P* value
Ask the pharmacist about the patient’s disease for dietetic consultation	35.3%	66.7%	0.059
Ask the pharmacist about the patient’s medication for dietetic consultation	41.2%	83.3%	0.012
Ask the pharmacist about the patient’s laboratory test data for dietetic consultation	24.2%	41.7%	0.254
Refer to medication record prepared by pharmacists during dietetic consultation	51.5%	75.0%	0.158
A pharmacist suggests/requests a dietetic consultation by a registered dietitian for patients	51.5%	100.0%	0.003
Report the contents of dietetic consultation to pharmacist after the consultation	54.5%	75.0%	0.215

**Table 7:** Concerns based on the state of utilization of occupational abilities

Item	Not able to fully utilize occupational abilities	Able to utilize occupational abilities	*P* value
It is difficult to find opportunity for dietetic consultation.	81.1%	41.7%	0.009
It is difficult to collect basic information about patients.	43.2%	16.7%	0.097
It is difficult to provide information to patients in an easy-to-understand manner.	72.2%	66.7%	0.714
It is difficult to choose the source for information provision.	35.1%	41.7%	0.683
It is difficult to set nutritional goals for patients.	67.6%	50.0%	0.273
It is difficult to handle questions by patients	78.4%	75.0%	0.807
There is not enough time to conduct dietetic consultation.	51.4%	25.0%	0.111
Need to obtain more knowledge on medications.	75.7%	83.3%	0.581
Need to obtain more knowledge on diseases.	97.3%	100.0%	0.565
Need to obtain more knowledge on health foods and supplements.	94.6%	91.7%	0.713

## Discussion

In the present study, we conducted a questionnaire survey for registered dietitians in community pharmacies, pharmacists in community pharmacies with registered dietitians/dietitians, and pharmacists in community pharmacies without registered dietitians/dietitians, in order to understand the actual status of dietetic consultation conducted by registered dietitians and the status of cooperation between registered dietitians and pharmacists.

Registered dietitians tended to provide dietetic consultation more frequently and had better explanatory skills compared to pharmacists. Specifically, registered dietitians provided dietetic consultation significantly more often for obese and hypertensive patients. Registered dietitians also provided dietetic consultation significantly more often than pharmacists in community pharmacies without registered dietitians/dietitians for diabetic patients. Registered dietitians had a significantly higher ability than pharmacists to explain about the nutritional and dietary factors that need attention for patients with kidney disease not requiring dialysis, hyperuricemia, gout, obesity, and hypertension. It has been reported that community pharmacies can contribute to weight management [10, 11]. Our study suggests that both the ability to explain about obesity and the frequency of dietetic consultation about obesity by registered dietitians were higher than those by pharmacists. Thus, registered dietitians, who are expert in nutritional management, may be able to provide better advice about weight management in community pharmacies.

The present study revealed that pharmacists in community pharmacies with registered dietitians/dietitians had greater perceptions of the need for nutritional and dietary management in community pharmacies than pharmacists in community pharmacies without registered dietitians/dietitians. Moreover, cooperation with registered dietitians/dietitians appeared to be related to the perceptions of the importance of pharmacists’ nutritional and dietary management. Some pharmacists that had acknowledged the merit of having registered dietitians/dietitians in community pharmacies thought that registered dietitians gave patients accurate guidance and were able to give more specific and detailed explanations than pharmacists, and while pharmacists could only provide general dietary guidance, registered dietitians were able to provide advice that was easy to incorporate into daily life. The results suggested that these pharmacists placed a high value on registered dietitians/dietitians’ expertise in nutritional and dietary management.

Pharmacists recognized the importance of nutritional and dietary management in community pharmacies and hoped that registered dietitians would play an active role in community pharmacies, while most of the registered dietitians considered that they were not able to utilize their occupational abilities sufficiently. In the “Able to utilize occupational abilities” group, the percentage of registered dietitians who answered “I ask the pharmacist about the patient’s medication for dietetic consultation” or “A pharmacist suggests/requests a dietetic consultation by a registered dietitian for patients”, was significantly higher. In the “Not able to fully utilize occupational abilities” group, the percentage of registered dietitians who felt “It is difficult to find opportunity for dietetic consultation” was significantly higher. These results suggested that cooperation between registered dietitians and pharmacists is related to the performance of registered dietitians. If the pharmacist determines that dietetic consultation is necessary, the pharmacist should collaborate with the registered dietitian, and the registered dietitian should proactively provide dietetic consultation. In addition, some registered dietitians felt that they have no time to provide sufficient consultation while they are busy with other work. Currently, there are many community pharmacies where registered dietitians also work as dispensing clerks. The present study suggested that in order to promote the activities of registered dietitians, it would be necessary to improve the environment within community pharmacies, such as lightening the registered dietitians’ burden of pharmacy office work.

The present survey shows that many dietetic consultations were triggered by patients’ requests, suggesting that pharmacy users’ needs for dietetic consultations are high. It has been reported that pharmacy users in the UK were less likely to recognize community pharmacies as a place to seek advice on nutrition, although UK community pharmacies offer advice about weight management [12]. In Japan, pharmacy users’ awareness of the health support functions of community pharmacies was also low [13]. In order to encourage people to use community pharmacies for dietetic consultation, it would be necessary to increase public awareness of the importance of health promotion activities in community pharmacies. In this study, we examined the recognition of the need for nutritional management in community pharmacies among registered dietitians and pharmacists (belonging to the same pharmacy group). There may be a bias in the responses, because the survey was conducted in pharmacies operated by a single company. In particular, most of the registered dietitians in this study had limited experience. In the pharmacies included in this study, newly graduated registered dietitians underwent a one-year initial training period prior to on-site dietetic consultation. It is possible that these factors may have influenced the results concerning the registered dietitians’ explanatory skills and demonstration of registered dietitians’ professional skills. In Japan, relatively few community pharmacies have registered dietitians. Therefore, this survey on the actual situation of dietetic consultation by registered dietitians may be helpful in planning for the future of community pharmacies. In addition, our study only focused on the perspectives of registered dietitians and pharmacists regarding nutritional management in community pharmacies. To promote the activities of registered dietitians in community pharmacies, it will also be important to investigate the perspectives of patients or potential users of community pharmacies.

## Conclusions

Our study is the first to clarify the actual status of dietetic consultation in community pharmacies from the perspective of registered dietitians in Japan. Our findings suggest that registered dietitians have a greater explanatory ability than pharmacists concerning nutritional and dietary management for patients with diseases. Therefore, it would be helpful for pharmacists to take more interest in the nutritional condition of patients, and to cooperate with registered dietitians by asking them to provide dietetic consultation. Moreover, cooperation between registered dietitians and pharmacists was related to pharmacists’ awareness of the importance of dietetic consultation. Placing registered dietitians in pharmacies will raise pharmacists’ awareness of nutritional and dietary management and will help community pharmacies play a greater role in disease prevention and health support in a community-based integrated care system.

## Supplementary Information

**Additional file 1.** English translations of the Japanese language questionnaires used for each type of participant. There are three types of questionnaires for registered dietitians, pharmacists with registered dietitians/dietitians and pharmacists without registered dietitians/dietitians. The results in our manuscript are based on the answers to some of these questions.

## Data Availability

The data that support the findings of this study are available from the corresponding author upon reasonable request. The data are not publicly available due to privacy or ethical restrictions.
