# Supplementary material for: Questionnaire survey investigation of the present status of dietetic consultation at community pharmacies from the perspectives of registered dietitians and pharmacists
Source: BMC Health Serv Res. 2021 Sep 8;21:935. doi: 10.1186/s12913-021-06959-3 (PMC8425041; doi:10.1186/s12913-021-06959-3)
Supplement: Supplementary file 1 — Additional file 1. English translations of the Japanese language questionnaires used for each type of participant. There are three types of questionnaires for registered dietitians, pharmacists with registered dietitians/dietitians and pharmacists without registered dietitians/dietitians. The results in our manuscript are based on the answers to some of these questions. [file 12913_2021_6959_MOESM1_ESM.docx]

Additional file

These are three types of questionnaires for registered dietitians, pharmacists with registered dietitians/dietitians and pharmacists without registered dietitians/dietitians. The questionnaire surveys were conducted in Japanese. The results in our manuscript are based on the answers to some of these questions. English translations of the questionnaires are shown below.

<Questionnaire surveys for registered dietitians>

**A. About dietetic consultations sessions**

**Question A1．Have you conducted dietetic consultation in dietetic consultations sessions?**

1．No　→　Go on to the section B: “About ordinary dietetic consultations”

2．Yes

**Question A2．How many years have passed after the first dietetic consultation in dietetic cunsultations session ?**

1．Less than 1 year　　→ Go on to the section B: “About ordinary dietetic consultations”

2．1 year or more – less than 3 years

3．3 years or more – less than 5 years

4．5 years or more – less than 10 years

5. 10 years or more

**Question A3．How many dietetic consultation sessions per year do you have on average? In addition, how many consultations in one consultation session do you have on average?**

Dietetic consultation sessions:　　　　　 per year

　Consultation sessions in one consultation session:　　　 per 1 session

**Question A4．How often do you conduct dietetic consultations for users of the following genders and ages in dietetic consultation sessions?**

|  | Never | Rarely | Neither | Sometimes | Often |
| --- | --- | --- | --- | --- | --- |
| Male (less than 30 years) | 1 | 2 | 3 | 4 | 5 |
| Male (30 years – 44 years) | 1 | 2 | 3 | 4 | 5 |
| Male (45 years – 64 years) | 1 | 2 | 3 | 4 | 5 |
| Male (65 years – 74 years) | 1 | 2 | 3 | 4 | 5 |
| Male (75 years or more) | 1 | 2 | 3 | 4 | 5 |
| Female (less than 30 years) | 1 | 2 | 3 | 4 | 5 |
| Female (30 years – 44 years) | 1 | 2 | 3 | 4 | 5 |
| Female (45 years – 64 years) | 1 | 2 | 3 | 4 | 5 |
| Female (65 years – 74 years) | 1 | 2 | 3 | 4 | 5 |
| Female (75 years or more) | 1 | 2 | 3 | 4 | 5 |

**Question A5． For each relationship with person who came to you for consultation in dietetic consultation sessions, please select the only one that best describes the frequency with which you receive consultation.**

|  | Never | Rarely | Neither | Sometimes | Often |
| --- | --- | --- | --- | --- | --- |
| ①The person oneself | 1 | 2 | 3 | 4 | 5 |
| ②Parents | 1 | 2 | 3 | 4 | 5 |
| ③Brothers or Sisters | 1 | 2 | 3 | 4 | 5 |
| ④Wife or Husband | 1 | 2 | 3 | 4 | 5 |
| ⑤Children | 1 | 2 | 3 | 4 | 5 |
| If you experience dietetic consultations from other persons except above ① to ⑤, please describe the details in parentheses. Ex) friends, colleagues, etc. | | | | | |
| Others  （ 　　　　　　　　　　） | 1 | 2 | 3 | 4 | 5 |

**Question A6．How often do you conduct the following action when you provide dietetic consultations in dietetic consultation sessions?**

|  | Never | Rarely | Neither | Sometimes | Often |
| --- | --- | --- | --- | --- | --- |
| Collect the basic information (medical history, medication, lifestyle, etc) of targets | 1 | 2 | 3 | 4 | 5 |
| Provide information by using some documents | 1 | 2 | 3 | 4 | 5 |
| Determine the goals which the target can complete | 1 | 2 | 3 | 4 | 5 |
| Introduce the next dietetic consultation session | 1 | 2 | 3 | 4 | 5 |
| Reserve the next dietetic consultation session | 1 | 2 | 3 | 4 | 5 |
| Make a document about the content of the consultation after the consultation | 1 | 2 | 3 | 4 | 5 |

**Question A7．What do you think are the issues about dietetic consultations in dietetic consultation sessions?**

|  |
| --- |

**Question A8．About Question A7, what do you think are the solutions to such issues?**

|  |
| --- |

**B．About ordinary dietetic consultations**

**Question B1．Have you conducted ordinary dietetic consultations?**

1．No　→　Go on to the section C: “Perspectives about dietetic consultations”

2．Yes

**Question B2．About the timing and/or situation that led to dietetic consultation, please select all the following settings you have experienced. (choose all)**

1. A patient consulted at the time of presenting prescription

2. A patient consulted while waiting for medication

3. A patient consulted at the time of payment

4. A visitor who did not have a prescription consulted

5. Registered dietitian approached the patient

6. Pharmacist recommended the patient to consult registered dietitian

7. Others (

**Question B3．How many ordinary dietetic consultations per 1 month do you have on average?**

**per 1 month**

**Question B4．How long do you conduct one ordinary dietetic consultation? Please choose the one in which you conduct most often.**

1. Less than 5 minutes

2. 5 minutes or more – less than 15 minutes

3. 15 minutes or more – less than 30 minutes

4. 30 minutes or more

**Question B5．How often do you conduct ordinary dietetic consultations for users of the following genders and ages?**

|  | Never | Rarely | Neither | Sometimes | Often |
| --- | --- | --- | --- | --- | --- |
| Male (less than 30 years) | 1 | 2 | 3 | 4 | 5 |
| Male (30 years – 44 years) | 1 | 2 | 3 | 4 | 5 |
| Male (45 years – 64 years) | 1 | 2 | 3 | 4 | 5 |
| Male (65 years – 74 years) | 1 | 2 | 3 | 4 | 5 |
| Male (75 years or more) | 1 | 2 | 3 | 4 | 5 |
| Female (less than 30 years) | 1 | 2 | 3 | 4 | 5 |
| Female (30 years – 44 years) | 1 | 2 | 3 | 4 | 5 |
| Female (45 years – 64 years) | 1 | 2 | 3 | 4 | 5 |
| Female (65 years – 74 years) | 1 | 2 | 3 | 4 | 5 |
| Female (75 years or more) | 1 | 2 | 3 | 4 | 5 |

**Question B6: For each relationship with person who came to you for ordinary dietetic consultation, please select the only one that best describes the frequency with which you receive consultation.**

|  | Never | Rarely | Neither | Sometimes | Often |
| --- | --- | --- | --- | --- | --- |
| ①The person oneself | 1 | 2 | 3 | 4 | 5 |
| ②Parents | 1 | 2 | 3 | 4 | 5 |
| ③Brothers or Sisters | 1 | 2 | 3 | 4 | 5 |
| ④Wife or Husband | 1 | 2 | 3 | 4 | 5 |
| ⑤Children | 1 | 2 | 3 | 4 | 5 |
| If you experience dietetic consultations from other persons except above ① to ⑤, please describe the details in parentheses. Ex) friends, colleagues, etc. | | | | | |
| Others  （ 　　　　　　　　　　） | 1 | 2 | 3 | 4 | 5 |

**Question B7．How often do you conduct the following action when you provide ordinary dietetic consultations?**

|  | Never | Rarely | Neither | Sometimes | Often |
| --- | --- | --- | --- | --- | --- |
| Collect the basic information (medical history, medication, lifestyle, etc) of targets | 1 | 2 | 3 | 4 | 5 |
| Provide information by using some documents | 1 | 2 | 3 | 4 | 5 |
| Determine the goals which the target can complete | 1 | 2 | 3 | 4 | 5 |
| Introduce the next dietetic consultation session | 1 | 2 | 3 | 4 | 5 |
| Reserve the next dietetic consultation session | 1 | 2 | 3 | 4 | 5 |
| Make a document about the content of the consultation after the consultation | 1 | 2 | 3 | 4 | 5 |

**Question B8．What do you think are the issues about ordinary dietetic consultations?**

|  |
| --- |

**Question B9．About Question B8, what do you think are the solutions to such issues?**

|  |
| --- |

**C．Perspectives about dietetic consultations**

**Question C1. Please select the most appropriate frequency of dietetic consultation for patients with various diseases.**

|  | Never | Rarely | Neither | Sometimes | Often |
| --- | --- | --- | --- | --- | --- |
| Kidney disease not requiring dialysis | 1 | 2 | 3 | 4 | 5 |
| Kidney disease in need of dialysis | 1 | 2 | 3 | 4 | 5 |
| Liver disease | 1 | 2 | 3 | 4 | 5 |
| Diabetes | 1 | 2 | 3 | 4 | 5 |
| Peptic ulcer | 1 | 2 | 3 | 4 | 5 |
| Anemia | 1 | 2 | 3 | 4 | 5 |
| Dyslipidemia | 1 | 2 | 3 | 4 | 5 |
| Hyperuricemia, gout | 1 | 2 | 3 | 4 | 5 |
| Heart disease | 1 | 2 | 3 | 4 | 5 |
| Obesity | 1 | 2 | 3 | 4 | 5 |
| Hypertension | 1 | 2 | 3 | 4 | 5 |
| Constipation | 1 | 2 | 3 | 4 | 5 |
| Diarrhea | 1 | 2 | 3 | 4 | 5 |
| Cancer | 1 | 2 | 3 | 4 | 5 |

**Question C2．How much ability do you have to explain about nutrition and dietary intake for each disease?**

|  | Never | Not very well | Neither | Somewhat | Very well |
| --- | --- | --- | --- | --- | --- |
| Kidney disease not requiring dialysis | 1 | 2 | 3 | 4 | 5 |
| Kidney disease in need of dialysis | 1 | 2 | 3 | 4 | 5 |
| Liver disease | 1 | 2 | 3 | 4 | 5 |
| Diabetes | 1 | 2 | 3 | 4 | 5 |
| Peptic ulcer | 1 | 2 | 3 | 4 | 5 |
| Anemia | 1 | 2 | 3 | 4 | 5 |
| Dyslipidemia | 1 | 2 | 3 | 4 | 5 |
| Hyperuricemia, gout | 1 | 2 | 3 | 4 | 5 |
| Heart disease | 1 | 2 | 3 | 4 | 5 |
| Obesity | 1 | 2 | 3 | 4 | 5 |
| Hypertension | 1 | 2 | 3 | 4 | 5 |
| Constipation | 1 | 2 | 3 | 4 | 5 |
| Diarrhea | 1 | 2 | 3 | 4 | 5 |
| Cancer | 1 | 2 | 3 | 4 | 5 |

**Question C3. Where do you provide dietetic consultation most frequently?**

1. On the bench or the chair in the pharmacy

2. At the counter for medication guidance

3. At a dedicated counter for dietetic consultation

4. Others ( )

**Question C4．Please select the most appropriate degree of concerns about the state of utilization of occupational abilities.**

|  | Strongly disagree | Disagree a little | Neither | Agree a little | Strongly agree |
| --- | --- | --- | --- | --- | --- |
| It is difficult to find opportunity for dietetic consultation. | 1 | 2 | 3 | 4 | 5 |
| It is difficult to collect basic information about patients. | 1 | 2 | 3 | 4 | 5 |
| It is difficult to provide information to patients in an easy-to-understand manner. | 1 | 2 | 3 | 4 | 5 |
| It is difficult to choose the source for information provision. | 1 | 2 | 3 | 4 | 5 |
| It is difficult to set nutritional goals for patients. | 1 | 2 | 3 | 4 | 5 |
| It is difficult to handle questions by patients | 1 | 2 | 3 | 4 | 5 |
| There is not enough time to conduct dietetic consultation. | 1 | 2 | 3 | 4 | 5 |
| Need to obtain more knowledge on medications. | 1 | 2 | 3 | 4 | 5 |
| Need to obtain more knowledge on diseases. | 1 | 2 | 3 | 4 | 5 |
| Need to obtain more knowledge on health foods and supplements. | 1 | 2 | 3 | 4 | 5 |

**Question C5．Please select the most appropriate choice about the frequency of dietetic consultations.**

1．Too little

2．Just the right frequency

3．Too frequent

**Question C6. (Only for registered dietitians that have conducted dietetic consultations in dietetic consultations sessions) Please select the most appropriate degree of concerns about the items below related to dietetic consultations sessions.**

|  | Strongly disagree | Disagree a little | Neither | Agree a little | Strongly agree |
| --- | --- | --- | --- | --- | --- |
| It is difficult to monitor the status patients continuously. | 1 | 2 | 3 | 4 | 5 |
| It is difficult to finish the dietetic consultation within the allotted time. | 1 | 2 | 3 | 4 | 5 |
| It is difficult to introduce the dietetic consultation for patients waiting for medication who did not reserve dietetic consultation. | 1 | 2 | 3 | 4 | 5 |

**D．About cooperation with pharmacists**

**Question D1．Have you ever felt that it is essential to cooperate between registered dietitians and pharmacists in dietetic consultations?**

1．No

2．Yes

**Question D2．Please select the most appropriate frequency of interactions between registered dietitians and pharmacists for each items.**

|  | Never | Rarely | Neither | Sometimes | Often |
| --- | --- | --- | --- | --- | --- |
| Ask the pharmacist about the patient’s disease for dietetic consultation | 1 | 2 | 3 | 4 | 5 |
| Ask the pharmacist about the patient’s medication for dietetic consultation | 1 | 2 | 3 | 4 | 5 |
| Ask the pharmacist about the patient’s laboratory test data for dietetic consultation | 1 | 2 | 3 | 4 | 5 |
| Refer to medication record prepared by pharmacists during dietetic consultation | 1 | 2 | 3 | 4 | 5 |
| A pharmacist suggests/requests a dietetic consultation by a registered dietitian for patients | 1 | 2 | 3 | 4 | 5 |
| Report the contents of dietetic consultation to pharmacist after the consultation | 1 | 2 | 3 | 4 | 5 |

**E．Work in community pharmacies as registered dietitians**

**Question E1．Do you think how much you utilize your occupational abilities in community pharmacies?**

1. Not at all
2. Not very much
3. Neither
4. Somewhat
5. Very well

**Question E2．What would you like to see improved in your work as a registered dietitian at a community pharmacy?**

|  |
| --- |

**Question E3．Please describe your most memorable dietetic consultation while working as a registered dietitian in a community pharmacy.**

|  |
| --- |

**F．About yourself**

**Question F1．Age**

1．20’s　　2．30’s　　3．40’s 4．50’s　　5．60’s　　6．70 years or more

**Question F2．Gender**

1．Male　　2．Female

**Question F3．Years of experience as a registered dietitian**

1．Less than 1 year

2．1 year or more – less than 3 years

3．3 years or more – less than 5 years

4．5 years or more – less than 10 years

5．10 years or more

**Question F4．Experience working in other facilities as a registered dietitian**

1. None

2. Hospital

3. Drugstore

4. Industrial food service facilities

5. Child Welfare Facilities

6. Nursing care facilities

7. Social welfare facilities / correctional facilities

8. School

9. Companies (food-related)

10. Companies (not food-related)

11. Others (　　　　　　　　　　　　　　　　　　　　　　　　　　　　　)

**Question F5. Qualifications other than registered dietitian**

1. None

2. Registered sales persons

3. Medical clerk

4. Pharmacy clerk

5. Others (　　　　　　　　　　　　　　　　　　　　　　　　　　)

**Question F6. Which of the following applies to you?**

1. Registered dietitian who graduated from a registered dietitian training facility

2. Registered dietitian who graduated from a dietitian training facility and had a practical experience

**Finally, please describe your opinions or impression about this survey if you have any.**

|  |
| --- |

This is the end of the questionnaire.

Thank you very much for your cooperation.

<Questionnaire surveys for pharmacists with registered dietitians/dietitians>

**A. About dietetic consultations sessions**

**Question A1．Do you conduct dietetic consultations sessions in your community pharmacy?**

1．No　→　Go on to the section B: “Perspectives about dietetic consultations”

2．Yes

**Question A2．How many dietetic consultation sessions do you have on average?**

**Ex) 2 times per 1 month, 1 time per 3 months**

　　　　　 per month(s)

**Question A3. How do you inform visitors to the community pharmacy about dietetic consultation session? Please select all that you are implementing.**

1. Posting posters in the pharmacy

2. Putting leaflets in the pharmacy

3. Giving leaflets at the time of medication guidance

4. Putting flags or signboards in front of the pharmacy

5. Posting on the web page of the pharmacy

6. Pharmacists’ recommendation directly to patients

7. Registered dietitians’ or dietitians’ recommendation directly to patients

8. Others (　　　　　　　　　　　　　　　　　　　　　　　　　　　　　)

**B．Perspectives about dietetic consultations**

**Question B1. Please select the most appropriate frequency of dietetic consultation by yourself (as a pharmacist) for patients with various diseases.**

|  | Never | Rarely | Neither | Sometimes | Often |
| --- | --- | --- | --- | --- | --- |
| Kidney disease not requiring dialysis | 1 | 2 | 3 | 4 | 5 |
| Kidney disease in need of dialysis | 1 | 2 | 3 | 4 | 5 |
| Liver disease | 1 | 2 | 3 | 4 | 5 |
| Diabetes | 1 | 2 | 3 | 4 | 5 |
| Peptic ulcer | 1 | 2 | 3 | 4 | 5 |
| Anemia | 1 | 2 | 3 | 4 | 5 |
| Dyslipidemia | 1 | 2 | 3 | 4 | 5 |
| Hyperuricemia, gout | 1 | 2 | 3 | 4 | 5 |
| Heart disease | 1 | 2 | 3 | 4 | 5 |
| Obesity | 1 | 2 | 3 | 4 | 5 |
| Hypertension | 1 | 2 | 3 | 4 | 5 |
| Constipation | 1 | 2 | 3 | 4 | 5 |
| Diarrhea | 1 | 2 | 3 | 4 | 5 |
| Cancer | 1 | 2 | 3 | 4 | 5 |

**Question B2．How much ability do you have to explain about nutrition and dietary intake for each disease?**

|  | Never | Not very well | Neither | Somewhat | Very well |
| --- | --- | --- | --- | --- | --- |
| Kidney disease not requiring dialysis | 1 | 2 | 3 | 4 | 5 |
| Kidney disease in need of dialysis | 1 | 2 | 3 | 4 | 5 |
| Liver disease | 1 | 2 | 3 | 4 | 5 |
| Diabetes | 1 | 2 | 3 | 4 | 5 |
| Peptic ulcer | 1 | 2 | 3 | 4 | 5 |
| Anemia | 1 | 2 | 3 | 4 | 5 |
| Dyslipidemia | 1 | 2 | 3 | 4 | 5 |
| Hyperuricemia, gout | 1 | 2 | 3 | 4 | 5 |
| Heart disease | 1 | 2 | 3 | 4 | 5 |
| Obesity | 1 | 2 | 3 | 4 | 5 |
| Hypertension | 1 | 2 | 3 | 4 | 5 |
| Constipation | 1 | 2 | 3 | 4 | 5 |
| Diarrhea | 1 | 2 | 3 | 4 | 5 |
| Cancer | 1 | 2 | 3 | 4 | 5 |

**Question B3. Please select the most appropriate frequency of the items below about dietetic consultation.**

|  | Never | Rarely | Neither | Sometimes | Often |
| --- | --- | --- | --- | --- | --- |
| You (as a pharmacist) are asked to advise on diet or nutrition by patients | 1 | 2 | 3 | 4 | 5 |
| You feel the need for dietetic consultation during medication guidance | 1 | 2 | 3 | 4 | 5 |
| You (as a pharmacist) conduct dietetic consultations for patients. | 1 | 2 | 3 | 4 | 5 |

**C．About cooperation with registered dietitians/dietitians**

**Question C1. Have you ever felt that it is essential to cooperate between registered dietitians and pharmacists in dietetic consultations?**

1．No

2．Yes

**Question C2．Please select the most appropriate frequency of interactions between pharmacists and registered dietitians/dietitians for each items.**

|  | Never | Rarely | Neither | Sometimes | Often |
| --- | --- | --- | --- | --- | --- |
| Consult with registered dietitians/dietitians about dietary management for patients. | 1 | 2 | 3 | 4 | 5 |
| Consult with registered dietitians/dietitians about health foods or supplements for patients | 1 | 2 | 3 | 4 | 5 |
| Consult with registered dietitians/dietitians about interactions between medication and diet | 1 | 2 | 3 | 4 | 5 |
| Offer the patient dietetic consultation by registered dietitians/dietitians | 1 | 2 | 3 | 4 | 5 |
| View records about dietetic consultation by registered dietitians/dietitians | 1 | 2 | 3 | 4 | 5 |
| Ask the registered dietitians/dietitians about the patient who took dietetic consultations by them. | 1 | 2 | 3 | 4 | 5 |

**Question C3．How much do you agree with the following items about nutritional and dietary management in community pharmacies?**

|  | Strongly disagree | Disagree a little | Neither | Agree a little | Strongly agree |
| --- | --- | --- | --- | --- | --- |
| Nutritional and dietary management at pharmacy is necessary. | 1 | 2 | 3 | 4 | 5 |
| Nutritional and dietary management at pharmacy improves patients' QOL. | 1 | 2 | 3 | 4 | 5 |
| Nutritional and dietary management at pharmacy improves the patient’s laboratory test data such as blood pressure or blood glucose. | 1 | 2 | 3 | 4 | 5 |
| Nutritional and dietary management at pharmacy lead to reducing the number of medications patients use. | 1 | 2 | 3 | 4 | 5 |
| The number of pharmacies with registered dietitians will increase in the future. | 1 | 2 | 3 | 4 | 5 |

**Question C4. Have you ever felt that registered dietitians/dietitians are helpful in the community pharmacy?**

1．No

2．Yes

**Question C5. (Only for persons who answered “2. Yes” in the question C3.) In what situations did you fell that registered dietitians/dietitians are helpful in the community pharmacy?**

|  |
| --- |

**Question C6. Please describe challenges for registered dietitians/dietitians in community pharmacies.**

|  |
| --- |

**D．About your pharmacy**

**Question D1. Percentages of prescriptions from a specific medical institution.**

1. Less than 70%

2. 70％ or more – less than 90%

3. 90% or more

**Question D2．The site of your pharmacy**

1. Near the clinic

2. Near the hospital that has 20 or more beds.

3. Near the hospital that has 200 or more beds.

4. In the medical mall

5. In the living area

6. Others（　　　　　　　　　　　　　　　　　　　　　　　　　　）

**Question D3．The number of prescriptions per 1 month**

1. less then 500

2. 500 or more – less than 1000

3. 1000 or more – less than 1500

4. 1500 or more – less than 2000

5. 2000 or more

**Question D4. The number of employees in the pharmacy (only workers at least 4 days a week)**

　　　　 Pharmacist

　　　　 Registered dietitian

　　　　 Dietitian

　　　　 Others

**Question D5. Is the pharmacy a health support pharmacy?**

1. Yes

2. No (We plan to apply for health support pharmacy status in the future.)

3. No (We DO NOT plan to apply for health support pharmacy status in the future.)

**E. About your pharmacy equipment**

**Question E1. How many places does your pharmacy have counters at which pharmacists provide information to the patient by face-to-face?**

1. Zero

2. One

3. Two

4. Three

5. Four

6. Five or more

**Question E2. Are there a chair on which patients can sit at the counter?**

1．No

2．Yes

**Question E3. How many people can sit in the waiting space?**

1. Zero

2. 1 - 3

3. 4 - 6

4. 7 - 9

5. 10 or more

**Question E4. Are there any places at which pharmacists or registered dietitians/dietitians can provide information to the patient except benches or chairs at the counter or in the waiting space?**

1．No

2．Yes（Which place?：　　　　　　　　　　　　 　　　　　）

**F．About yourself**

**Question F1．Age**

1．20’s　　2．30’s　　3．40’s 4．50’s　　5．60’s　　6．70 years or more

**Question F2．Gender**

1．Male　　2．Female

**Question F3．Years of experience as a registered dietitian**

1．Less than 1 year

2．1 year or more – less than 3 years

3．3 years or more – less than 5 years

4．5 years or more – less than 10 years

5．10 years or more

**Question F4．Experience working in other facilities**

1. None

2. Hospital

3. Drugstore

4. Pharmaceutical drug company

5. Non-pharmaceutical drug company

6. Contract Research Organization

7. Independent Administrative Agency under the jurisdiction of Public Offices

8. government worker

9. Others (　　　　　　　　　　　　　　　　　　　　　　　　　　　　　　　)

**Finally, please describe your opinions or impression about this survey if you have any.**

|  |
| --- |

This is the end of the questionnaire.

Thank you very much for your cooperation.

<Questionnaire surveys for pharmacists without registered dietitians/dietitians>

**A. About dietetic consultations sessions**

**Question A1．Do you conduct dietetic consultations sessions in your community pharmacy?**

1．No　→　Go on to the section B: “Perspectives about dietetic consultations”

2．Yes

**Question A2．How many dietetic consultation sessions do you have on average?**

**Ex) 2 times per 1 month, 1 time per 3 months**

　　　　　 per month(s)

**Question A3. How do you inform visitors to the community pharmacy about dietetic consultation session? Please select all that you are implementing.**

1. Posting posters in the pharmacy

2. Putting leaflets in the pharmacy

3. Giving leaflets at the time of medication guidance

4. Putting flags or signboards in front of the pharamacy

5. Posting on the web page of the pharmacy

6. Pharmacists’ recommendation directly to patients

7. Others (　　　　　　　　　　　　　　　　　　　　　　　　　　　　　)

**B．Perspectives about dietetic consultations**

**Question B1. Please select the most appropriate frequency of dietetic consultation by yourself(as a pharmacist) for patients with various diseases.**

|  | Never | Rarely | Neither | Sometimes | Often |
| --- | --- | --- | --- | --- | --- |
| Kidney disease not requiring dialysis | 1 | 2 | 3 | 4 | 5 |
| Kidney disease in need of dialysis | 1 | 2 | 3 | 4 | 5 |
| Liver disease | 1 | 2 | 3 | 4 | 5 |
| Diabetes | 1 | 2 | 3 | 4 | 5 |
| Peptic ulcer | 1 | 2 | 3 | 4 | 5 |
| Anemia | 1 | 2 | 3 | 4 | 5 |
| Dyslipidemia | 1 | 2 | 3 | 4 | 5 |
| Hyperuricemia, gout | 1 | 2 | 3 | 4 | 5 |
| Heart disease | 1 | 2 | 3 | 4 | 5 |
| Obesity | 1 | 2 | 3 | 4 | 5 |
| Hypertension | 1 | 2 | 3 | 4 | 5 |
| Constipation | 1 | 2 | 3 | 4 | 5 |
| Diarrhea | 1 | 2 | 3 | 4 | 5 |
| Cancer | 1 | 2 | 3 | 4 | 5 |

**Question B2．How much ability do you have to explain about nutrition and dietary intake for each disease?**

|  | Never | Not very well | Neither | Somewhat | Very well |
| --- | --- | --- | --- | --- | --- |
| Kidney disease not requiring dialysis | 1 | 2 | 3 | 4 | 5 |
| Kidney disease in need of dialysis | 1 | 2 | 3 | 4 | 5 |
| Liver disease | 1 | 2 | 3 | 4 | 5 |
| Diabetes | 1 | 2 | 3 | 4 | 5 |
| Peptic ulcer | 1 | 2 | 3 | 4 | 5 |
| Anemia | 1 | 2 | 3 | 4 | 5 |
| Dyslipidemia | 1 | 2 | 3 | 4 | 5 |
| Hyperuricemia, gout | 1 | 2 | 3 | 4 | 5 |
| Heart disease | 1 | 2 | 3 | 4 | 5 |
| Obesity | 1 | 2 | 3 | 4 | 5 |
| Hypertension | 1 | 2 | 3 | 4 | 5 |
| Constipation | 1 | 2 | 3 | 4 | 5 |
| Diarrhea | 1 | 2 | 3 | 4 | 5 |
| Cancer | 1 | 2 | 3 | 4 | 5 |

**Question B3. Please select the most appropriate frequency of below items about dietetic consultation.**

|  | Never | Rarely | Neither | Sometimes | Often |
| --- | --- | --- | --- | --- | --- |
| You (as a pharmacist) are asked to advise on diet or nutrition by patients | 1 | 2 | 3 | 4 | 5 |
| You feel the need for dietetic consultation during medication guidance | 1 | 2 | 3 | 4 | 5 |
| You (as a pharmacist) conduct dietetic consultations for patients. | 1 | 2 | 3 | 4 | 5 |

**C．About the nutritional and dietary management in community pharmacies**

**Question C1．How much do you agree with the following items about nutritional and dietary management in community pharmacies?**

|  | Strongly disagree | Disagree a little | Neither | Agree a little | Strongly agree |
| --- | --- | --- | --- | --- | --- |
| Nutritional and dietary management at pharmacy is necessary. | 1 | 2 | 3 | 4 | 5 |
| Nutritional and dietary management at pharmacy improves patients' QOL. | 1 | 2 | 3 | 4 | 5 |
| Nutritional and dietary management at pharmacy improves the patient’s laboratory test data such as blood pressure or blood glucose. | 1 | 2 | 3 | 4 | 5 |
| Nutritional and dietary management at pharmacy lead to reducing the number of medications patients use. | 1 | 2 | 3 | 4 | 5 |
| The number of pharmacies with registered dietitians will increase in the future. | 1 | 2 | 3 | 4 | 5 |

**Question C2. Would you like to place registered dietitians/dietitians in your pharmacy?**

**1.** Yes

2. No

**Question C3. Please describe the reason for your answer to question C2.**

|  |
| --- |

**D．About your pharmacy**

**Question D1. Percentages of prescriptions from a specific medical institution.**

1. Less than 70%

2. 70％ or more – less than 90%

3. 90% or more

**Question D2．The site of your pharmacy**

1. Near the clinic

2. Near the hospital that has 20 or more beds.

3. Near the hospital that has 200 or more beds.

4. In the medical mall

5. In the living area

6. Others（　　　　　　　　　　　　　　　　　　　　　　　　　　）

**Question D3．The number of prescriptions per 1 month**

1. less then 500

2. 500 or more – less than 1000

3. 1000 or more – less than 1500

4. 1500 or more – less than 2000

5. 2000 or more

**Question D4. The number of employees in the pharmacy (only workers at least 4 days a week)**

　　　　 Pharmacist

　　　　 Others

**Question D5. Is the pharmacy a health support pharmacy?**

1. Yes

2. No (We plan to apply for health support pharmacy status in the future.)

3. No (We DO NOT plan to apply for health support pharmacy status in the future.)

**E. About your pharmacy equipment**

**Question E1. How many places does your pharmacy have counters at which pharmacists provide information to the patient by face-to-face?**

1. Zero

2. One

3. Two

4. Three

5. Four

6. Five or more

**Question E2. Are there a chair on which patients can sit at the counter?**

1．No

2．Yes

**Question E3. How many people can sit in the waiting space?**

1. Zero

2. 1 - 3

3. 4 - 6

4. 7 - 9

5. 10 or more

**Question E4. Are there any places at which pharmacists or registered dietitians/dietitians can provide information to the patient except benches or chairs at the counter or in the waiting space?**

1．No

2．Yes（Which place?：　　　　　　　　　　　　 　　　　　）

**F．About yourself**

**Question F1．Age**

1．20’s　　2．30’s　　3．40’s 4．50’s　　5．60’s　　6．70 years or more

**Question F2．Gender**

1．Male　　2．Female

**Question F3．Years of experience as a registered dietitian**

1．Less than 1 year

2．1 year or more – less than 3 years

3．3 years or more – less than 5 years

4．5 years or more – less than 10 years

5．10 years or more

**Question F4．Experience working in other facilities**

1. None

2. Hospital

3. Drugstore

4. Pharmaceutical drug company

5. Non-pharmaceutical drug company

6. Contract Research Organization

7. Independent Administrative Agency under the jurisdiction of Public Offices

8. government worker

9. Others (　　　　　　　　　　　　　　　　　　　　　　　　　　　　　　　)

**Finally, please describe your opinions or impression about this survey if you have any.**

|  |
| --- |

This is the end of the questionnaire.

Thank you very much for your cooperation.
